# Whey Protein Hydrolysate Enhances HSP90 but Does Not Alter HSP60 and HSP25 in Skeletal Muscle of Rats

**DOI:** 10.1371/journal.pone.0083437

**Published:** 2014-01-20

**Authors:** Carolina Soares Moura, Pablo Christiano Barboza Lollo, Priscila Neder Morato, Luciana Hisayama Nisishima, Everardo Magalhães Carneiro, Jaime Amaya-Farfan

**Affiliations:** 1 Department of Food and Nutrition, Faculty of Food Engineering, University of Campinas (UNICAMP), Campinas, São Paulo, Brazil; 2 Institute of Biology (IB), University of Campinas (UNICAMP), São Paulo, Brazil; Universidad Pablo de Olavide, Centro Andaluz de Biología del Desarrollo-CSIC, Spain

## Abstract

Whey protein hydrolysate (WPH) intake has shown to increase HSP70 expression. The aim of the present study was to investigate whether WPH intake would also influences HSP90, HSP60 and HSP25 expression, as well as associated parameters. Forty-eight male Wistar rats were divided into sedentary (unstressed) and exercised (stressed) groups, and were fed with three different sources of protein: whey protein (WP), whey protein hydrolysate (WPH) and casein (CAS) as a control, based on the AIN93G diet for 3 weeks. WPH intake increased HSP90 expression in both sedentary and exercised animals compared to WP or CAS, however no alteration was found from exercise or diet to HSP60 or HSP25. Co-chaperone Aha1 and p-HSF1 were also increased in the exercised animals fed with WPH in comparison with WP or CAS, consistent with enhanced HSP90 expression. VEGF and p-AKT were increased in the WPH exercised group. No alteration was found in BCKDH, PI3-Kinase (p85), GFAT, OGT or PGC for diet or exercise. The antioxidant system GPx, catalase and SOD showed different responses to diet and exercise. The data indicate that WPH intake enhanced factors related to cell survival, such as HSP90 and VEGF, but does not alter HSP60 or HSP25 in rat skeletal muscle.

## Introduction

Heat shock proteins (HSPs) were discovered by Feruccio Ritossa in 1962 following observation of the chromosomes of *Drosophila melanogaster* that were submitted to heat shock treatment. HSPs are a complex physiological defense mechanism [1] that confer higher tolerance and cell resistance against a variety of aggressor agents, show a strong cytoprotective effect [2], favor the maintenance of cell integrity and structure and may promote cell survival during periods of stress. They may repair damaged proteins or facilitate their degradation when the damage is irreversible [1], [3]. Heat shock proteins may be grouped into families that are classified according to their molecular weight, such as HSP90, HSP70, HSP60 and HSP25 [1]. The process of HSPs synthesis involves heat shock factor (HSF1), which is phosphorylated and upon reaching the nucleus, binds to the gene promoter that synthesizes these proteins.

The increase in HSPs content has been shown to provide cytoprotection to skeletal muscle against stress from exercise and some forms of muscle damage [4], [5] and it is believed that the development of new strategies and procedures that may increase the expression of HSPs would be of practical relevance [6].

Whey protein (WP) represents approximately 20% of the proteins present in bovine milk and has been recognised for its high nutritive value, high digestibility and fast absorption. In particular, whey protein hydrolysate (WPH), has shown several important features, such as protective effect against oxidative stress, demonstrating its antioxidant capacity [7], reduction of muscle damage markers in humans [8], increased glycogen and GLUT4 translocation [9], and an anti-stress effect [10], [11].

We have recently shown, for the first time, that the intake of whey protein hydrolysate (WPH) increases the exercise-induced HSP70 expression in several tissues [10]. The aim of the present study was to investigate whether WPH intake also influences the expression of other HSPs and their pathway, as well as parameters associated with cell survival, such as the vascular endothelial growth factor (VEGF).

## Materials and Methods

### Animals

Forty-eight male Wistar rats (specific-pathogen free) from the Multidisciplinary Center for Biological Research (University of Campinas, SP, Brazil) were maintained under controlled conditions (temperature: 22°C, humidity 55%, reverse 12-hour light/dark cycle) in individual growth cages with access to commercial feed (Labina, Purina, Brazil) and water *ad libitum*, until they reached 150 g of body mass. The Ethics Committee on Animal Experimentation of the University of Campinas approved all experimental procedures (CEEA-UNICAMP, protocol 2297-1).

### Experimental diets and procedures

The diets were based on the AIN93-G diet [12], except that the protein content was 12% [10] and whey protein (WP), whey protein hydrolysate (WPH) or casein (CAS, control) was the only protein source used. Table 1 shows the diet formulation and the amino acid profile of the protein sources. When the animals reached 150 g (±5.2) of body mass, they were randomly assigned to six groups (n = 8), corresponding to the three diets (CAS, WP and WPH) and two exercise regimes: Sedentary (unstress) and exercised (stressed). The diets were consumed for 3 weeks.

**Table 1:** Formulation of the diets (g/kg of diet) and amino acid profile of the protein sources.

Item	CAS	WP	WPH
**Diet Composition**			
Corn Starch	437.92	427.31	425.00
Dextrinised starch	145.42	141.90	141.13
Sucrose	110.16	107.50	106.92
WPH	-------	-------	156.41
WP	-------	152.77	-------
CAS	135.96	-------	-------
Vegetable oil	70.00	70.00	70.00
Fiber (cellulose)	50.00	50.00	50.00
Mineral mixture	35.00	35.00	35.00
Vitamin mixture	10.00	10.00	10.00
L-Cystine	3.00	3.00	3.00
Choline bitartrate	2.50	2.50	2.50
Tert-butylhydroquinone	0.014	0.014	0.014
**Amino acid profile**			
Aspartate	5.96	11.52	11.16
Glutamate	19.00	18.82	17.99
Serine	4.68	5.31	5.04
Glycine	1.39	1.74	1.75
Histidine	2.12	1.31	1.27
Arginine	3.03	2.66	2.31
Threonine	3.56	7.64	7.40
Alanine	2.30	5.11	4.89
Proline	8.85	5.89	5.68
Tyrosine	4.57	2.88	2.78
Methionine	2.32	2.51	2.52
Cystine	0.16	1.48	1.60
Isoleucine	4.51	6.88	6.97
Leucine	7.62	10.14	10.15
Valine	5.36	5.68	5.81
Phenylalanine	3.89	2.86	2.78
Lysine	6.62	9.20	9.48

### Exercise protocol

The animals in the exercised groups were subjected to five exercise sessions on a treadmill at the speed of 22 m/min for 30 minutes in the last week of treatment (last five days). Exercise on a treadmill is known to be an effective way to promote HSP response and has been adopted by researchers for this purpose [10], [13]. After the last exercise session, the rats were allowed to recover for 6 hours for maximal HSP expression, and were then killed by decapitation [14].

### Western blotting

The gastrocnemius sample (200 mg) was homogenized in 5 volumes of buffer (200 mM EDTA (Sigma 03685), pH 7.0, 1M Tris Base (Bio-Rad #161-0719), pH 7.5, 10 mM orthovanadate (Sigma S6508), 2 mM phenylmethanesulfonyl fluoride (Sigma P7626), 10 mM sodium pyrophosphate (Sigma 221368), 0.1 mg/mL aprotinin (Sigma 10820), 100 mM sodium fluoride (Sigma 71519), Triton 10% (Sigma #019K0151), ultrapure water) using Polytron (Pro Scientific model Pro 200) and centrifuged (Sigma, model 2K15, number serial 57707, Germany) at 14,000 g for 40 minutes at 4°C and the supernatant was collected. The total protein content was determined in the supernatant using the Lowry method [15]. The samples were treated with Laemmli buffer containing dithiothreitol (DTT) (Bio-Rad #161-0611). After heating samples at 95°C for 5 min, the proteins were subjected to SDS-PAGE (8%) and transferred using a semi-dry system (Bio-Rad, CA, USA) to a nitrocellulose membrane of 0.22 µM (Bio-Rad, cat. 162-0112). A molecular weight standard was used and run concurrently on each gel for accurate determination of the proper molecular weight for each antibody (Thermo Scientific, #26634). The nitrocellulose membranes were treated with blocking buffer (3% nonfat dried milk or albumin, 10 mmol/L Tris Base (Bio-Rad #161-0719), 150 mmol/L NaCl (Sigma 71379), and 0.02% Tween 20 (Sigma P1379).

The membranes were incubated with the appropriate primary antibodies overnight to assess the protein level of: HSP90 (Stressgen, Victoria, BC, Canada; Ref. ADI-SPA 831 diluted 1∶3000, MW90 kDa), HSP60 (Stressgen, Victoria, BC, Canada; Ref. ADI-SPA 806 diluted 1∶2000, MW60 kDa), HSP25 (Stressgen, Victoria, BC, Canada; Ref. ADI-SPA 801 diluted 1∶2000, MW25 kDa), Aha1 (Abcam, Cambridge, Ref. ab83036 diluted 1∶2000, MW38 kDa), SOD (Abcam, Cambridge, Ref. ab51254 diluted 1∶10.000, MW18 kDa), Catalase (Santa Cruz, CA, USA, Ref. sc271803 diluted 1∶1000, MW55 kDa), GPx (Abcam, Cambridge, Ref. ab22604 diluted 1∶2000 MW22 kDa), p-HSF1 phosphorylated in serine 230 (Santa Cruz, CA, USA, Ref. sc30443 diluted 1∶1000, MW90 kDa), HSF (Stressgen, Victoria, BC, Canda; Ref. SPA 950 diluted 1∶500), GFAT (Santa Cruz, CA, USA, Ref. sc134894 diluted 1∶1000, MW77 kDa), OGT (Abcam, Cambridge, Ref. ab59135 diluted 1∶1000, MW110 kDa), GAPDH (Stressgen, Victoria, BC, Canada, Ref. ADI 905734 diluted 1∶1000), VEGF (Abcam, Cambridge, Ref. ab46154 diluted 1∶2000, MW43 kDa), BCKDH (Abcam, Cambridge, Ref. ab59747 diluted 1∶2000, MW46 kDa), p-AKT phosphorylated in serine 473 (Santa Cruz, CA, USA, Ref. sc7985-R diluted 1∶1000, MW56 kDa), AKT (Santa Cruz, CA, USA, Ref. sc8312 diluted 1∶1000, MW56 kDa), PI 3-Kinase (p85), N-SH2 domain (catalog number #06-496, Upstate Biotechnology NY, USA diluted 1∶1000) and PGC (Abcam, Cambridge, Ref. ab72230 diluted 1∶1000, MW110 kDa). The appropriate secondary antibodies were used for detection. The bands were visualized using a UVITEC Cambridge instrument (model Alliance LD2). The blots were quantified using the digital program UVITEC.

### Determination of amino acids of the protein sources and muscle samples

The protein sources (dry basis) were hydrolysed at 110°C in 6 M HCl (Merck 1003171000) for 24 hours. The hydrolysed samples were then diluted in deionized water, α-aminobutyric (Sigma 162663) acid was added as the internal standard, and the amino acids were derivatized with phenylisothiocyanate (Sigma P1034). The gastrocnemius free amino acids were extracted with 80/20 (v/v) methanol and HCL 0.1 M prior to derivatization. For neutral amino acids and basic amino acid was used standard from Sigma (A6407; A6282 respectively). The PTH-derivatives were chromatographed using a Luna C-18, 100 Å; 5 µm, 250×4.6 mm (00G-4252-EQ) column at 50°C, detected at 254 nm [16]. The retention times were (min): aspartate 3.04, glutamate 3.37, hydroxyproline 4.62, asparagine 5.90, serine 6.21, glutamine 6.65, glycine 6.86, histidine 8.0, taurine 9.32, arginine 9.81, threonine 10.87, alanine 11.70, proline 13.39, tyrosine 32.56, valine 37.18, methionine 40.51, cysteine 47.67, isoleucine 49.17, leucine 49.89, phenylalanine 56.08, tryptophan 59.0, lysine 61.08.

### Blood sample collection

Blood samples were collected at sacrifice and centrifuged at 3000×*g* (4°C, 15 min) to obtain the serum. Analyses of serum for uric acid, creatine kinase (CK), lactate dehydrogenase (LDH), aspartate aminotransferase (AST), alanine aminotransferase (ALT), total protein, albumin, creatinine and urea were carried out using a clinical kits (Laborlab, São Paulo, Brazil) with spectrophotometric determination (Beckman-Coulter DU640, CA, USA). Glucose in the blood was measured using an Accu-Chek Active glucometer (Roche Diagnostics, Mannheim, Germany).

### Data analysis

The data were analyzed by ANOVA, followed by the Duncan post-hoc test, using SPSS (Statistical Package for the Social Sciences, Chicago, United States) software version 17.0. The level for significance was set to *p<0.05*.

## Results

### HSP90, HSP60, HSP25, Aha1 and p-HSF1

**Figure 1:**
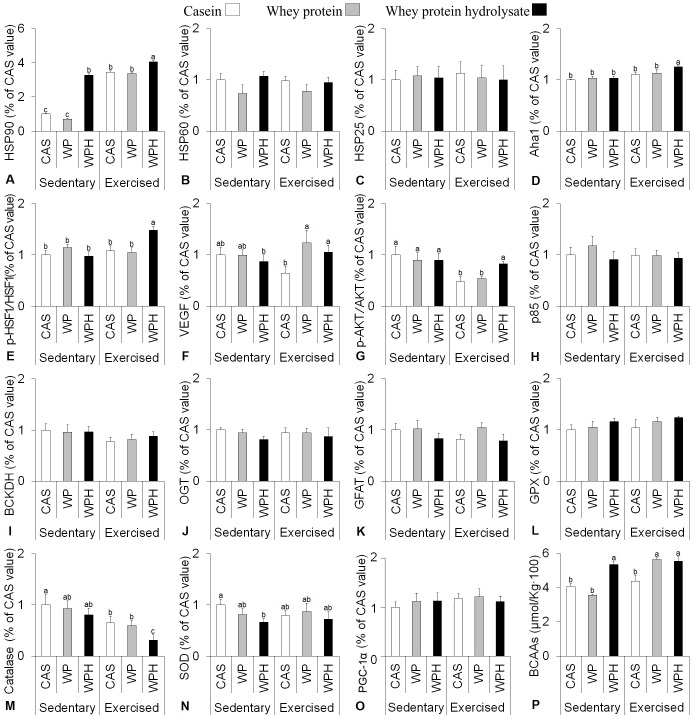
Exercised groups were subjected to five sessions on a treadmill at the speed of 22/min for 30 minutes during the last week of the diet treatment. The data were obtained from gastrocnemius collected 6(n = 8 per group). The dietary proteins were consumed for 3 weeks. Mean and standard error for the Western blot analysis of: HSP90 (A), HSP60 (B), HSP25 (C), Aha1 (D), p-HSF1 (E), VEGF (F), p-AKT (G), PI3 Kinase (p85) (H), BCKDH (I), OGT (J), GFAT (K), GPx (L), Catalase (M), SOD (N), PGC (O), Sum of BCAAs (P). Protein sources: whey protein (WP), whey protein hydrolysate (WPH), Casein (CAS) control. Six groups: Sedentary CAS (control), Sedentary WP, Sedentary WPH, Exercised CAS, Exercised WP and Exercised WPH. All values were compared and related to CAS sedentary (protein source control) and reported as %CAS value. Different letters represent significant differences.

**Figure 2:**
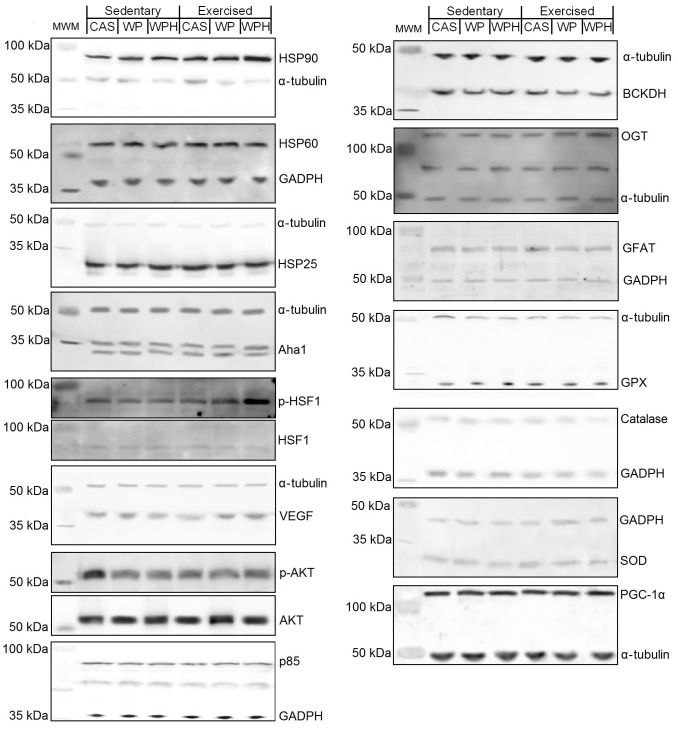
Exercised groups were subjected to five sessions on a treadmill at the speed of 22/min for 30 minutes during the last week of the diet treatment. The data were obtained from gastrocnemius collected 6(n = 8 per group). The dietary proteins were consumed for 3 weeks. Mean and standard error for the blood analysis of: Albumin (A), total protein (B), uric acid (C), glucose (D), LDH (E), CK (F), AST (G), ALT (H), urea (I), creatinine (J). Protein sources: whey protein (WP), whey protein hydrolysate (WPH), Casein (CAS) control. Six groups: Sedentary CAS (control), Sedentary WP, Sedentary WPH, Exercised CAS, Exercised WP and Exercised WPH. All values were compared and related to CAS sedentary (protein source control) and reported as %CAS value. Different letters represent significant differences.

Whey protein hydrolysate (WPH) intake promoted an increase in HSP90 expression in sedentary and exercised animals, compared to WP and CAS. No effect due diet or exercise was observed on HSP60 or HSP25 expression, as illustrated in Figure 1 (A, B, C), respectively and Figure 2.

Co-chaperone activator of heat shock protein ATPase (Aha1) expression followed the elevation found in HSP90 promoted by WPH intake when compared to casein (control) or whey protein (WP) in exercised animals (Figure 1 – D). Figure 1 (E) shows that there was increase in phosphorylated heat shock factor (p-HSF1) expression, leading to HSF1 activation, caused by WPH intake in exercised animals.

### VEGF, p-AKT, PI3-Kinase and BCKDH

Vascular endothelial growth factor (VEGF) increased in exercised animals that consumed WPH and WP compared to CAS. No difference was observed between the diets in sedentary animals, as shown in Figure 1 (F). The data show that WPH intake in the exercised group increased phosphorylated protein kinase B (p-AKT) expression when compared to casein and whey protein; however, no alteration was observed in the sedentary group for the different protein sources (Figure 1 G). No effect of diet or exercise was observed on phosphatidyl inositol kinase (PI3-kinase/p85) or branched-chain α-keto acid dehydrogenase (BCKDH) (Figure 1H and 1I respectively).

### OGT and GFAT - Hexosamine biosynthetic pathway

O-β-acetylglucosaminyltransferase (OGT) and glutamine fructose-6-amidotransferase (GFAT), key proteins of the hexosamine biosynthetic pathway were not affected by the different protein sources or exercise, as illustrated in Figure 1 (J, K), respectively and Figure 2.

### GPx, SOD and catalase – Antioxidant system

**Figure 3:**
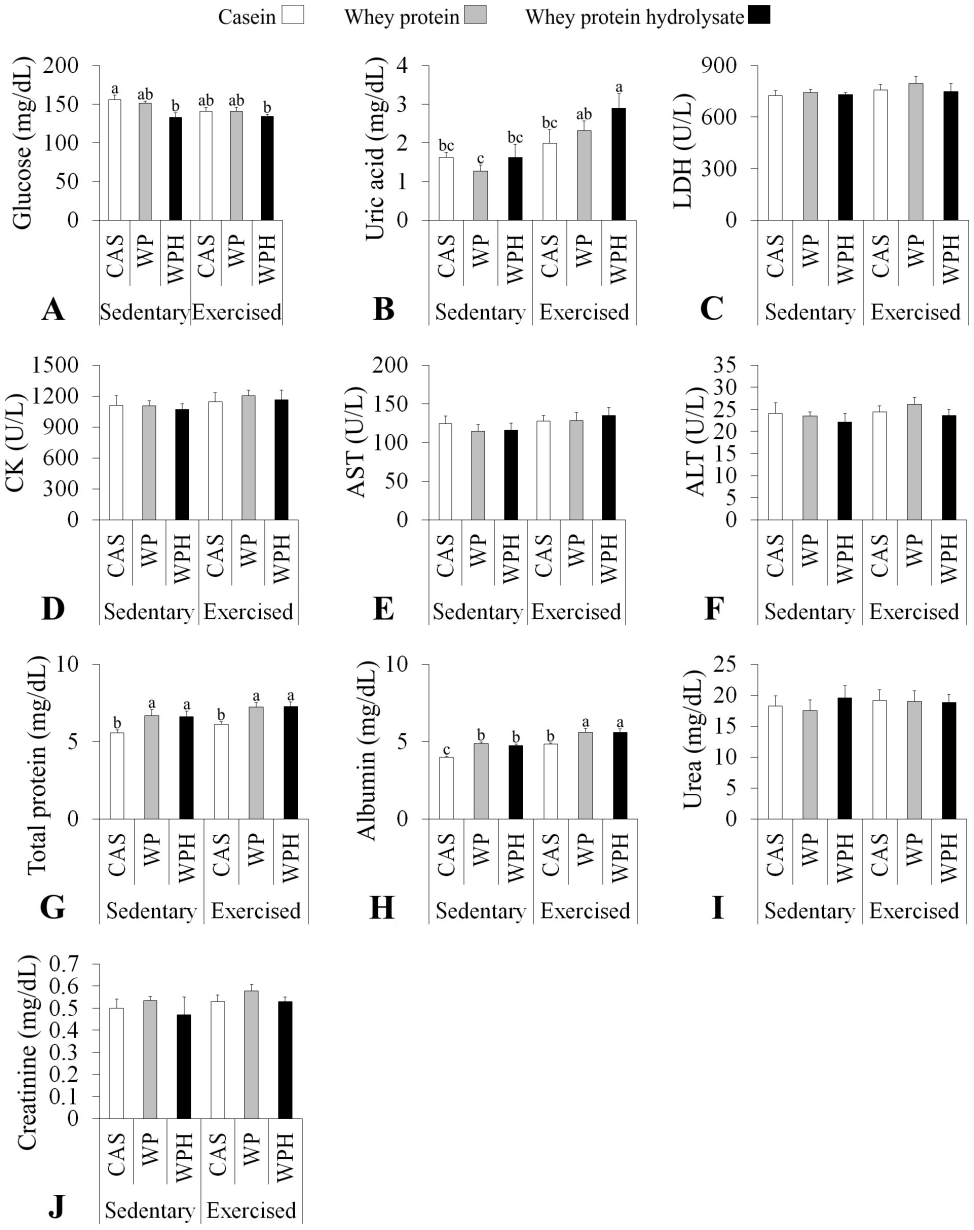
Representative blots of independent experiments are shown. GAPDH or α tubulin is also shown as loading control.

There was no difference in the expression of glutathione peroxidase (GPx), according to Figure 1 (L). Catalase showed a progressive reduction from sedentary animals to exercised animals (Figure 1 M). Superoxide dismutase (SOD) was reduced in sedentary animals that consumed WPH in comparison with WP and CAS. However, there was no difference between the protein sources in the exercised group (Figure 1 N). Diet and exercise had no effect on peroxisome proliferator-activated receptor-γ coactivator (PGC 1α) (Figure 1 O and Figure 3).

### Dietary intake and weight parameters

Table 2 shows that the consumption of the different protein sources did not affect daily food consumption and did not differ in their effects on weight gain, protein efficiency ratio (PER) or food efficiency coefficient (FEC). Furthermore, none of the protein sources altered organ weight, which remained normal.

**Table 2:** Dietary intake and organs weight parameters.

	Sedentary						Exercised					
	CAS		WP		WPH		CAS		WP		WPH	
	Mean	SEM	Mean	SEM	Mean	SEM	Mean	SEM	Mean	SEM	Mean	SEM
Diet intake/day	23.40	0.76	22.70	0.49	24.89	0.63	24.9	1.12	22.83	1.08	23.45	0.90
Protein intake	53.42	4.21	56.13	6.30	58.63	4.00	63.79	7.02	54.14	5.10	56.82	3.36
PER^1^	4.21	0.09	4.08	0.07	4.17	0.07	3.80	0.04	4.21	0.06	4.16	0.07
Weight start	62.42	3.30	56.30	3.34	56.18	3.38	58.67	2.57	56.77	2.52	59.88	3.12
Body mass gain	224.95	9.14	229.35	8.18	244.83	5.16	242.94	8.93	228.37	6.81	236.47	5.99
FEC^2^	0.50	0.01	0.49	0.01	0.50	0.02	0.46	0.03	0.50	0.01	0.49	0.01
Heart *	0.36	0.01	0.36	0.01	0.34	0.01	0.35	0.01	0.37	0.02	0.37	0.01
Lung *	0.51	0.01	0.51	0.01	0.52	0.01	0.53	0.01	0.56	0.02	0.5	0.01
Spleen *	0.25	0.01	0.29	0.01	0.25	0.01	0.29	0.01	0.29	0.01	0.28	0.01
Kidney *	0.35	0.01	0.36	0.01	0.35	0.01	0.35	0.01	0.37	0.01	0.39	0.01
Gastrocnemius *	0.52	0.01	0.52	0.02	0.50	0.02	0.53	0.01	0.55	0.01	0.53	0.01
Soleus *	0.03	0.002	0.03	0.001	0.03	0.002	0.03	0.003	0.03	0.001	0.03	0.001

1 Protein efficiency ratio;

2 Food efficiency coefficient.

Diets: CAS – Casein (control), WP – Whey protein, WPH – Whey protein hydrolysate.

* tissue weight related to body weight (100 g).

### Blood analysis

The consumption of the different protein sources by the sedentary or the exercised group did not alter kidney, liver or muscle damage parameters, as illustrated in Figure 3 (E–J). With regard to albumin and total serum protein parameters (Figure 3 A, B), there was increase in the animals that received WP and WPH diets in either both sedentary and the exercised groups, compared to the casein diet. Uric acid was not affected by the protein source in the sedentary group. However, in the exercised group, WPH increased uric acid in comparison with casein. Exercise also increased the uric acid concentration, but only in the animals that consumed WP and WPH (Figure 3 C). Glucose was lower in the sedentary animals in the WPH group in comparison with casein (Figure 3 D).

### Muscle free amino acid profile

Previous work suggests the involvement of plasma free branched chain amino acids (BCAAs) in the enhancement of HSP70 [10] it was desirable to verify if there would be a similar effect in muscle. There was a significant difference in all free amino acids in the muscle (Table 3), with the exception of serine, histidine, methionine and phenylalanine. The intake of WP associated with exercise resulted in increased concentrations of valine. Isoleucine showed a different behavior, such that WPH intake increased its concentration in both the sedentary and exercised groups when compared to other protein sources (casein and WP). Exercise also increased isoleucine in all dietary groups. WPH increased leucine in the sedentary group, whereas in the exercised group both WP and WPH increased leucine. The sum of BCAAs (isoleucine, valine and leucine) for the different protein sources is illustrated in the Figure 1 (P).

**Table 3:** Mean muscle free amino acid profile (µmol/kg).

	Sedentary						Exercised					
Amino acid	CAS		WP		WPH		CAS		WP		WPH	
	Mean	SEM	Mean	SEM	Mean	SEM	Mean	SEM	Mean	SEM	Mean	SEM
Aspartic acid	532.42^c^	42.76	1524.03^a^	1077.65	906.90^b^	325.00	375.42^c^	17.13	646.02^bc^	269.61	441.08^c^	42.93
Glutamate	2624.71^c^	480.19	4256.60^ab^	126.96	4035.48^ab^	1549.37	2576.68^c^	99.54	4681.69^a^	699.52	3225.35^bc^	397.79
Hydroxyproline	158.64^bc^	46.98	249.64^a^	176.52	149.35^bc^	27.47	122.15^bc^	7.06	173.05^b^	1.74	94.78^c^	15.93
Asparagine	857.15^ab^	199.66	944.32^a^	87.34	874.02^ab^	170.18	664.58^bc^	9.20	919.84^a^	75.23	615.50^c^	90.54
Serine	2183.06	449.53	2117.05	234.78	1973.73	359.99	1817.59	115.28	2119.35	293.29	1672.45	60.15
Glutamine	8446.66^bc^	1522.09	10223.21^b^	172.63	9073.03^bc^	2046.33	7139.44^c^	99.72	12936.53^a^	461.69	7892.17^c^	557.05
Glycine	708.24^b^	63.25	869.51^a^	74.03	760.64^ab^	75.47	514.64^c^	24.37	744.21^ab^	89.08	525.45^c^	70.31
Histidine	281.92	79.71	200.30	8.20	262.83	58.63	218.94	29.43	198.35	109.05	160.98	25.15
Arginine	11988.93^c^	1922.66	15603.53^b^	11033.36	18411.99^a^	142.16	9607.97^d^	467.83	16896.30^ab^	1505.50	11950.89^c^	343.63
Taurine	3240.83^c^	407.41	4304.41^b^	134.75	5073.11^a^	8.47	2477.55^d^	19.39	4595.29^ab^	758.24	3345.59^c^	123.78
Threonine	2419.16^cd^	597.10	2976.74^abc^	184.50	3338.21^ab^	729.38	1993.04^d^	157.46	3674.79^a^	548.61	2564.9^bcd^	164.88
Alanine	978.16^cd^	404.07	1663.98^a^	11.00	1359.05^ab^	255.30	664.92^d^	12.88	1515.24^a^	83.34	1124.42^bc^	80.10
Proline	1035.64^a^	151.87	808.23^b^	16.14	908.52^ab^	21.87	939.99^ab^	49.42	811.20^b^	33.05	881.92^b^	30.33
Tyrosine	87.44^a^	22.40	54.24^b^	3.18	46.25^b^	5.01	88.71^a^	8.73	66.09^b^	8.10	49.72^b^	2.15
Valine	218.63^b^	40.14	175.23^b^	6.97	218.51^ab^	20.52	204.93^b^	35.65	258.97^a^	1.44	213.93^ab^	34.91
Methionine	98.62	26.08	89.65	10.62	95.19	7.63	78.25	6.73	99.97	18.88	94.95	1.48
Cystine	8.80^c^	1.00	12.30^b^	8.69	18.90^b^	0.26	48.73^a^	20.20	31.49^ab^	21.36	17.85^b^	0.68
Isoleucine	50.07^c^	6.79	48.18^c^	10.06	95.80^b^	9.05	93.46^b^	44.92	97.64^b^	7.18	141.36^a^	34.81
Leucine	138.45^b^	6.48	131.97^b^	2.95	220.82^a^	25.09	139.75^b^	21.76	209.07^a^	18.98	199.73^a^	4.76
Phenylalanine	63.79	3.32	41.89	26.43	67.32	15.35	59.02	1.42	55.63	3.89	67.90	12.27
Tryptophan	19.66^c^	3.63	39.06^ab^	3.06	33.76^b^	10.36	11.63^c^	3.61	47.58^a^	6.18	34.65^b^	10.20
Lysine	895.09^ab^	263.05	1159.80^a^	192.02	1197.71^a^	370.21	558.00^b^	67.22	897.17^ab^	136.76	979.09^ab^	233.35

## Discussion

We have recently reported that whey protein hydrolysate intake increases HSP70 expression in different rat tissues [10]. The aim of the present study was to investigate whether the intake of the same dietary proteins (whey protein concentrate and hydrolysate as well as casein) as a protein source in the diet influences the expression of other HSPs such as HSP90, HSP60 and HSP25 and parameters related to the HSP system.

WPH intake increased HSP90 expression in the skeletal muscle of rats in both the sedentary and exercised groups. HSP90 is a chaperone protein essential to the viability of eukaryotic cells and is abundantly expressed, representing about 1–2% of cell proteins even in unstressed cells [17], [18], while its concentration may increase in response to stressful situations [17]. This is consistent with our results for sedentary (unstressed cells) and exercised (stressed cells) groups. HSP90 is involved in preventing protein aggregation [18] in the folding process, in the maintenance of cell protein integrity and stabilization and activation of at least 200 signaling proteins [19].

According to the literature, increased HSP90 expression has a cytoprotective effect [4], promotes skeletal muscle protection in exercised animals [20], increases cell resistance [18], and protects against the accumulation of reactive oxygen species, thus reducing cytotoxicity [21]. Our results indicate that WPH intake favors the defense system represented by HSP90. However, no effect on HSPs was observed in the animals fed unhydrolyzed whey protein (WP), so this cannot be attributed to the speed of absorption, because this is the same (fast absorption) between the hydrolyzed and non-hydrolyzed forms of whey protein. It is possible that the hydrolyzed form contained bioactive peptides with the ability to enhance HSP expression.

Together with the increase in HSP90, WPH intake also promoted increased expression of its co-chaperone Aha1, which is known for accelerating conformational transitions and increasing the ATPase activity of HSP90 [22], thus facilitating the interaction between ATP and the N-terminal domain of HSP90, which is ATP-dependent [2]. Aha1 may also be regulated by stress, together with HSP90, and it is believed that Aha1 is involved in folding processes mediated by HSP90 [23].

Another significant result of this study was the increase in vascular endothelial growth factor (VEGF) expression promoted by WPH and WP intake associated with exercise. VEGF plays an important role in angiogenesis, which may favor the supply of oxygen and substrates in tissues. Furthermore, under stress conditions, VEGF may promote cell survival by activating the phosphatidyl inositol kinase (PI3-kinase-p85) and phosphorylating protein kinase B (AKT). Akt is involved in anti-apoptotic signaling and is capable of promoting survival [24]. In the present study, no effect was found on PI3-kinase (p85); however, WPH intake in exercised animals increased Akt phosphorylation.

There were no alterations in HSP60 expression which, according to the literature, increases slightly in stressful situations [2], the same was true for HSP25. For other kinds of stress, the HSP60 seems to be more responsive, such as in Alzheimer's disease [25].

HSP expression may be muscle fiber type specific. In the present study, we performed the analysis in the gastrocnemius muscle, which is a tissue with mixed fibers. Folkesson et al. [26] showed that the type of fiber (slow or fast) does not change the HSP60 expression. While HSP70 expression showed higher staining intensity in type I fibers compared to type II fibers. Hsp25 showed higher intensity in type II fibers compared to type I fibers. Milne and Noble [27] show that the soleus muscle (slow fibers) is more stress responsive compared to fast muscle fibers and that this response is dependent on the intensity of the exercise.

Heat shock factor (HSF1) plays an important role in the transcription process of HSPs because it is rapidly activated and is present in most eukaryotes in several types of stress. Under stress conditions, HSF1 assumes an active trimeric state, which allows it to reach the nucleus where it is phosphorylated and binds to the gene promoter, initiating HSP synthesis [1], [3], [18]. There are other HSFs such as HSF2 and HSF3, however the function and capacity of HSF1 in HSP synthesis cannot be compensated for by other heat shock factors [28]. WPH promoted an increase in HSF1 phosphorylation in exercised animals, consistent with increased HSP90 expression.

It is known that the amino acid glutamine is capable of increasing the expression of different HSPs; however, the mechanism remains to be elucidated. *In vitro* studies have suggested that the key proteins GFAT and OGT from the hexosamine biosynthetic pathway could be involved [29]. We probed these proteins *in vivo*, investigating whether whey protein intake could also show some involvement on the hexosamine biosynthetic pathway, but no alterations were observed.

The antioxidant capacity of whey protein has already been described by others [7], [30]. In our experiment, exercise reduced catalase expression in the gastrocnemius muscle when compared to the sedentary group. These results are consistent with other reports [31], [32]. In contrast, some studies have demonstrated an increase in catalase in skeletal muscle after exercise [33], [34]. In human skeletal muscle, catalase levels measured on a daily basis from pre-exercise up to the sixth day after exercise (time course) did not show any tendency or linearity [5]. This controversial result was also found for SOD [32], [35], [36]. A time-course study showed that there was a significant reduction in SOD levels in skeletal muscle after the third day that continued to decline up to the sixth day after exercise [5]. In our study, GPx did not differ between groups, although a reduction in GPx levels after exercise has already been reported [37]. These results indicate that antioxidant enzymes in skeletal muscle may be modulated independently and differentially influenced by diet and exercise.

A possible role of BCAAs in the capacity of WPH to increase HSP70 expression was recently suggested [10]. In that study [10], we observed that the concentrations of free amino acids (isoleucine and leucine) in the plasma were increased in sedentary animals consuming a WPH diet compared to either casein or whey protein. The same finding was observed in the present study, but for muscle free amino acids. Despite the greater availability of BCAAs in the present study, there was no change in branched-chain α-keto acid dehydrogenase (BCKDH), which is responsible for BCAA catabolism in skeletal muscle [38].

The consumption of the different protein sources used in the present study did not alter liver and kidney blood parameters. Regarding uric acid, which is considered the most abundant and powerful serum antioxidant [39], our results indicate that the protein source may affect the antioxidant protection of uric acid caused by exercise. Whey proteins have been reported to preserve the levels of serum albumin and total proteins in sedentary animals, as well as during exercise [10], [40], consistent with our results.

In conclusion, the data obtained in the present study indicate that intake of whey protein hydrolysate enhanced factors related to cell survival, such as HSP90 and VEGF expression in the skeletal muscle of rats when compared to other dietary protein sources (WP and casein). There was no alteration in HSP60 or HSP25 expression caused by diet or exercise.
