# Correction: Whey Protein Hydrolysate Enhances HSP90 but Does Not Alter HSP60 and HSP25 in Skeletal Muscle of Rats

**DOI:** 10.1371/annotation/0cb4a83a-2b26-410f-bdda-ff62dae25eea

**Published:** 2014-01-30

**Authors:** Carolina Soares Moura, Pablo Christiano Barboza Lollo, Priscila Neder Morato, Luciana Hisayama Nisishima, Everardo Magalhães Carneiro, Jaime Amaya-Farfan

The titles and legends for Figure 2 and the title of Figure 3 are incorrect. The title and legend currently appearing with Figure 2 belong with Figure 3, and the title currently appearing with Figure 3 belongs with Figure 2. The Figure images are in correct order. 

In addition, the Funding statement is incorrect. The Funding statement should read: "This work was supported by FAPESP (Proc. 2011/13035-1, 2012/05859-7 and 2013/02862-0), CNPq and CAPES. The funders had no role in study design, data collection and analysis, decision to publish, or preparation of the manuscript."

